# Hydrogen Sulfide (H_2_S) Metabolism, Iron Overload, and Apoptosis–Autophagy Equilibrium in Vascular Smooth Muscle Cells

**DOI:** 10.3390/antiox14050560

**Published:** 2025-05-08

**Authors:** Hassan Mustafa Arif, Ming Fu, Rui Wang

**Affiliations:** 1 Department of Biology, York University, Toronto, ON M3J 1P3, Canada; harif@yorku.ca (H.M.A.); mingf1@yorku.ca (M.F.); 2 College of Basic Medicine, Shandong Second Medical University, Weifang 261053, China

**Keywords:** H_2_S biology, iron metabolism, iron–H_2_S interaction, smooth muscle cells, apoptosis, autophagy

## Abstract

Iron overload contributes to proliferative vascular diseases, yet its interplay with hydrogen sulfide (H_2_S) in vascular smooth muscle cell (VSMC) proliferation remains poorly understood. This study elucidates H_2_S’s role in mitigating iron-overload-induced oxidative stress and cellular damage. Using aortic VSMCs from wildtype (WT) and cystathionine γ-lyase-knockout (CSE-KO) mice treated with ferric ammonium citrate (FAC) at concentrations equivalent to serum levels of iron and citrate, we demonstrate that FAC triggers the integrated stress response (ISR) in WT cells, upregulating CSE to enhance H_2_S production. The ISR mediator ATF4 activates caspases but simultaneously induces CSE, which inhibits caspase activity and promotes autophagy via AMPK signaling. In CSE-KO cells, iron overload leads to diminished Ferritin upregulation, unchecked Caspase activation, and impaired autophagy compared to WT cells. Exogenous H_2_S restored iron homeostasis by enhancing Ferritin expression, activating NRF2 antioxidant pathways, and restoring apoptosis–autophagy equilibrium in both WT and KO cells. These findings establish H_2_S as a critical regulator of iron-induced VSMC dysfunction, highlighting its therapeutic potential in managing vascular pathologies linked to iron dysregulation.

## 1. Introduction

Iron participates in diverse cellular functions from oxygen transport to energy metabolism. These roles of iron are realized by its ferric (Fe^3+^) or ferrous (Fe^2+^) status and its binding strength with different macromolecules. For example, its weak conjugation with citrate yields the form of labile iron [1,2]. Diminished iron level is a key pathogenic factor for anemia. Iron overload is an abnormal condition of iron metabolism, where more iron is present than is physiologically required [3]. It contributes to numerous detrimental reactions, such as the Fenton reaction, to produce reactive oxygen species (ROS) that can cause widespread cellular damage [2]. Iron overload can be a direct complication of genetic diseases, such as heterozygous hemochromatosis and Friedreich ataxia, or secondary to diseases like thalassemia, where repeat blood transfusions are required [4]. As a comorbid factor in vascular diseases [2], excess iron exacerbates atherosclerosis with oxidative stress and apoptosis in vascular smooth muscle cells (VSMCs) [5].

Hydrogen sulfide (H_2_S) is a key gasotransmitter that is produced mainly via three enzymes, Cystathionine gamma-lyase (CSE), Cystathionine beta synthase, and 3-Mercaptopyruvate sulfurtransferase [2]. Recently, Cysteinyl-tRNA synthetases and the non-enzymatic reaction between cysteine and iron were also reported to produce significant quantities of H_2_S in cells [6]. The discovery that endogenous H_2_S can cause endothelium-dependent vasorelaxation [7] paved the way for scientists to explore the role of H_2_S in other physiological avenues. Endogenous H_2_S in the vasculature is primarily CSE-derived. Our previous studies have shown that CSE ablation exacerbates vascular complications like hypertension and atherosclerosis [8].

The interaction between iron and H_2_S in the vasculature has been investigated mostly using toxic levels of extracellular iron concentrations [9,10]. One study on the formation of iron–sulfur clusters or the formation of non-enzymatic H_2_S via iron and vitamin B6 used toxic doses, ranging from 50 to 100 µM, of ferric or ferrous iron [2,11,12]. In another study on H_2_S-facilitated conversion of iron regulatory protein 1 (IRP1) to its aconitase form in VSMCs, 100 µM of ferric chloride was used. The modulation of the expression and activity of the CSE/H_2_S system by iron at physiological or pathophysiologic levels has been unknown [2,11,12]. Whether and how H_2_S causes cellular iron overload without increasing extracellular iron to toxic levels is not clear.

While iron overload decreases autophagy and triggers cell death mainly via ferroptosis or apoptosis, depending on the cell type and the iron overload model [9,13], H_2_S has been shown to trigger apoptosis via the p38/MAPK and ERK pathways in VSMCs [14,15]. H_2_S has also been shown to regulate autophagy, since it can directly influence different autophagic proteins like adenosine monophosphate-activated protein kinase (AMPK), nuclear factor erythroid 2-related factor 2 (NRF2), and SIRT [16,17,18]. However, the integrated effects of iron overload and H_2_S on apoptosis and autophagy in VSMCs remain unexplored. This study aimed to investigate the interplay between iron overload and H_2_S in modulating apoptotic and autophagic pathways in VSMCs, providing new insights into their potential crosstalk in vascular pathophysiology.

## 2. Material and Methods

### 2.1. Animals and Protocols

All protocols were approved by the Animal Care Committee, York University. Briefly, wildtype mice with the background C57BL/6J x129SvEv and in-house created homozygous CSE-KO mice with the same genetic background were used for all the experiments. The homozygous CSE-KO mice were produced after backcrossing at least 10 generations of WT mice with heterozygous CSE-KO mice, as described in our previous study [7]. All mice had unlimited access to water and standard rodent chow ad libitum. They were kept in HVAC-ventilated cages on a 12:12 light/dark cycle.

### 2.2. Aortic VSMC Culture

Mouse aortic VSMCs were isolated as previously described, with modifications [15,19]. Aortae of 8-week-old male mice were excised and kept in ice-cold Kreb’s buffer with the following composition, in mM: 118 NaCl, 4.7 KCl, 1.18 KH_2_PO_4_, 1.17 MgSO_4_, 5 glucose, 25 NaHCO_3_, and 2.5 mM CaCl_2_. The dish containing an isolated aorta in Kreb’s buffer was kept on ice and continuously gassed with 95% oxygen and 5% CO_2_. A thin wire was inserted into the lumen to scrape and remove endothelial cells. Surrounding adipose tissue was carefully removed, and the aorta was placed in fresh 1.4 mg/mL collagenase type II solution prepared in serum-free Dulbecco’s Modified Eagle Medium (DMEM) for 20 min at 37 °C in a humidified 5% CO_2_ incubator. Supplementing DMEM with 2.05 g of NaHCO_3_ helped keep the pH of the DMEM at 7.4 in the incubator with 5% CO_2_. After 20 min of incubation, the adventitious layer was carefully rolled off the aorta, leaving only the medial layer containing VSMCs. The medial layer was incubated with fresh 1.4 mg/mL collagenase solution prepared in DMEM in a CO_2_ incubator for 4 h to release the VSMCs. Once sufficient cells were released, DMEM with 10% FBS was added to quench the collagenase, followed by centrifugation at 350× *g* for 10 min to pellet the VSMCs. The VSMCs were resuspended in DMEM containing 10% FBS and plated in a 35 mm culture dish. Cells were left undisturbed in the incubator for 5 days, when colonies of VSMCs started to appear. The medium was changed every 48 h until confluency was reached, which took 5 to 7 days. The presence of aortic VSMCs was confirmed by the expression of α-smooth muscle actin and the absence of platelet endothelial cell adhesion molecules.

Cells were passaged upon 80% confluency, and cells from passages 3–7 were used for the experiments. In all the experiments, cells were first synchronized by adding serum-free medium for 24 h, followed by the reintroduction of media with 10% FBS in the different treatment groups, which included phosphate-buffered saline (PBS) as a control, 0.1 mM ferric ammonium citrate (FAC), 0.1 mM NaHS, and cells pretreated with 0.1 mM NaHS for 4 h, followed by the addition of 0.1 mM FAC (N+F).

### 2.3. Iron Source

FAC obtained from MP Biomedicals, catalog number 158040, was used as an iron source for all the experiments. Because FAC contains 17% ferric iron, 0.1 mM of FAC is equivalent to 17 µM of iron. The average plasma iron concentration in C57BL6J mice is 17–23 µM, and the average serum iron concentration is 19–21 µM [2]. The average citrate concentration in mouse plasma is between 100 and 130 µM [20].

### 2.4. Cell Viability

Cell viability was assessed via the 3-(4,5-dimethylthiazol-2-yl)-2,5-diphenyltetrazolium bromide (MTT) assay, as previously described [19]. Cells were seeded with a density of 5000 cells per well in 96-well plates. After different treatments, MTT (5 mg/mL) was added, and the plates were incubated for 4 h at 37 °C in a 5% CO_2_ incubator. Dimethyl sulfoxide (DMSO) (200 µL) was added post-incubation for 20 min to dissolve the insoluble purple formazan. Plates were read on a Biotek Synergy HTX multimode plate reader (Agilent, Mississauga, ON, Canada) at 570 nm. Viability was expressed as the percentage change in absorbance (purple formazan) relative to controls of the same genotype (WT or CSE-KO).

### 2.5. Cell Proliferation

Cell proliferation was assessed using a colorimetric Bromodeoxyuridine (BrdU) assay kit (Calbiochem, Oakville, ON, Canada), as described previously [19]. Cells were seeded at a density of 5000 cells per well in a 96-well plate. After different treatments, the BrdU label was added for 4 h, after which fixing/denaturing reagents were added and incubated for 30 min. Anti-BrdU antibody was diluted 1:100 and added post-fixing/-denaturing for 1 h, followed by 3 washes with wash buffer provided with the kit. Peroxidase goat anti-mouse IgG HRP conjugate was added and incubated for 30 min, followed by 3 more washes. After the final wash, the substrate solution was mixed for 15 min, followed by the addition of a stop solution and measurement of absorbance on a Biotek Synergy HTX multimode plate reader (Agilent, Mississauga, ON, Canada) at 450/540 nm using 540 nm as background.

### 2.6. Ferrozine Assay

The intracellular iron content was measured via the ferrozine assay as described previously [21]. Cells were seeded at a density of 20,000 cells per well in a 24-well plate. After treatments, cells were lysed with fresh 200 µL iron-releasing agent (a mixture of 1.4 M HCl and 4.5% KMnO_4_, 1:1). The plates were sealed in aluminum foil and incubated at 60 °C for 2 h. Afterward, the plates were removed from the incubator and cooled to room temperature. Further, 30 µL iron detection reagent (6.5 mM ferrozine, 6.5 mM neocuproine, 2.5 mM ammonium acetate, and 1 M ascorbic acid) was added to each well for a 30 min reaction. The resulting absorbance was measured on a Biotek Synergy HTX multimode plate reader (Agilent, Mississauga, ON, Canada) using a 550 nm wavelength.

### 2.7. H_2_S Production Rate

The H_2_S production rate was measured via our previously described method [19]. In brief, lead acetate paper was prepared by soaking filter paper in 1% lead acetate for 1 h, then placing the filter paper in the oven to dry. Cell lysates prepared in ice-cold PBS with a final concentration of 100 µg protein in 50 µL were reacted with 10 µL of 10 mM cysteine and 10 μL of 2 mM pyridoxal 5′-phosphate in a 96-well plate overlaid with lead acetate filter paper. The 96-well plate was sealed in aluminum foil and placed in a 37 °C incubator for 2 h, after which the lead acetate filter paper was imaged and the resulting darkened, circular bands representing H_2_S activity were quantified using ImageJ software version 1.54h (NIH, Bethesda, MD, USA) [22].

### 2.8. Aconitase Activity Assay

Aconitase activity was measured using an aconitase assay kit (Abcam, Cambridge, MA, USA) as described previously [10]. Briefly, after treatment, cytoplasmic aconitase (c-aconitase) was isolated by homogenizing cell pellets in assay buffer, followed by centrifuging at 800× *g* for 10 min. The supernatant collected was centrifuged at 20,000× *g* for 15 min to pellet the mitochondrial aconitase (m-aconitase). The c-aconitase-containing supernatant was activated with 10 µL of activation solution per 100 µL supernatant and incubated on ice for 1 h. Afterward, 50 µL of the activated sample was incubated with 50 µL of the reaction mixture for 1 h at room temperature, and the remaining 50 µL of the activated sample was used to measure the background. After incubation, 10 µL of developing solution was added, and the resulting absorbance was measured at 450 nm on a Biotek Synergy HTX multimode plate reader (Agilent, Mississauga, ON, Canada).

### 2.9. ROS Production

ROS production was measured using CellROX^TM^ Green Reagent (Invitrogen, Burlington, ON, Canada). Cells were seeded with a density of 100,000 cells per 35 mm plate. After different treatments, CellRox reagent was added at a final concentration of 5 µM and incubated for 30 min at 37 °C. Plates were washed with PBS 3 times, and Hoechst 33342 was added as a counterstain, which was further incubated for 10 min. The plates were immediately imaged using an Olympus x71 fluorescence microscope (Olympus America Inc., Center Valley, PA, USA) with FITC (green) channels for ROS and DAPI (blue) channels for the Hoechst-stained nuclei. The fluorescence intensity ratio between the green and blue channels was calculated using ImageJ.

### 2.10. Lipid Peroxidation

Lipid peroxidation levels were measured using BODIPY581/591 C11 dye. Cells were seeded with a density of 100,000 cells per 35 mm plate. After different treatments, 2.5 µM BODIPY dye was added. Plates were put on an orbital shaker for 5 min to evenly distribute the dye and incubated in a 37 °C incubator with 5% CO_2_ for 20 min. The medium was removed, and PBS with Hoechst 33342 was added as a counterstain and further incubated for 10 min. The plates were immediately imaged using an Olympus x71 fluorescence microscope (Olympus America Inc., Center Valley, PA, USA) with FITC (green) channels for oxidized nuclei, TRITC (red) channels for the reduced probe, and DAPI (blue) channels to normalize against Hoechst-stained nuclei. The fluorescence intensity ratio between the red and green channels was calculated using ImageJ.

### 2.11. DNA Fragmentation (TUNEL) Assay

DNA fragmentation was assessed using a fluorescent FragEL DNA fragmentation detection kit (Calbiochem, Oakville, ON, Canada). Cells were seeded at a density of 100,000 cells per 35 mm plate containing a fibronectin-coated coverslip. After treatments, coverslips with cells were permeabilized with 20 µg/mL of proteinase K for 5 min, followed by 3 washes with Tris-buffered saline (TBS). Cells were equilibrated with 1X equilibration buffer for 15 min, then the equilibration buffer was removed and TdT was added to label the reaction mixture and incubated for 1 h. The reaction was terminated by washing 3 times with TBS. Coverslips were counterstained with Hoechst, mounted on slides, and visualized using an Olympus x71 fluorescence microscope (Olympus America Inc., Center Valley, PA, USA) with an FITC and DAPI channel. Mean fluorescence intensity was calculated using ImageJ.

### 2.12. Caspase 3/7 Activity

Cellular apoptosis was assessed by measuring caspase activity via CellEvent™ Caspase-3/7 Green ReadyProbes™ Reagent (Invitrogen, Burlington, ON, Canada). Cells were seeded with a density of 100,000 cells per 35 mm plate. After different treatments, 20 µL of dye was added to each plate and incubated in a 37 °C incubator with 5% CO_2_ for 10 min. Cells were visualized using an Olympus x71 fluorescence microscope (Olympus America Inc., Center Valley, PA, USA) with an FITC channel. Fluorescence intensity was calculated using ImageJ.

### 2.13. Autophagy Assay

Autophagy was assessed using the autophagy assay kit (MAK138, Sigma Aldrich, Oakville, ON, Canada), which produces fluorescence in DAPI channels when bound to autophagosomes. Cells were seeded at a density of 100,000 cells per 35 mm plate. After different treatments, cells were incubated with 1× autophagosome detection reagent for 1 h at 37 °C in an incubator with 5% CO_2_. Afterward, cells were washed 3 times with PBS and imaged immediately with an Olympus x71 fluorescence microscope (Olympus America Inc., Center Valley, PA, USA) with DAPI channels. The mean fluorescence intensity was calculated using ImageJ software.

### 2.14. Nuclear and Cytoplasmic Extraction

Nuclear and cytoplasmic extracts were separated using NE-PER reagent (ThermoScientific, Mississauga, ON, Canada). Harvested cells were centrifuged at 350× *g* for 5 min, and the supernatant was removed. The resulting cell pellet was resuspended in ice-cold 200 µL CER I reagent and vortexed for 15 s, followed by resting on ice for 10 min. Further, 11 µL of ice-cold CER II was added and vortexed twice for 5 s with 1 min intervals on ice. Lysate was centrifuged at maximum speed at 4 °C, and the supernatant containing cytoplasmic extract was transferred to a new tube. The resulting pellet with nuclear extract was resuspended in 100 µL ice-cold NER reagent.

### 2.15. Integrated Stress Response (ISR) Pathway Inhibition

ISR was inhibited using ISRIB (Sigma, Oakville, ON, Canada), a drug that inhibits phosphorylation of eukaryotic translation initiation factor 2 (p-eIF2α), and activating transcription factor 4 (ATF4) [23]. ISRIB stock was prepared in DMSO warmed to 65 °C and diluted in PBS, which was syringe-filtered prior to use. VSMCs were serum-starved overnight, followed by the reintroduction of DMEM with 10% FBS and 0.1 µM of ISRIB for 1 h. In the presence of ISRIB, either PBS (control) or 0.1 mM FAC (I+F group) was added to the culture medium for 48 h.

### 2.16. Western Blot Analysis

Cells were sonicated in ice-cold, fresh lysis buffer (1× RIPA buffer with 1% PMSF and 1% protease inhibitor cocktail). Lysates were separated via SDS-PAGE, transferred onto PVDF membranes, and incubated with a blocking buffer (5% milk or 5% BSA). Primary antibodies, including 1:1000 anti-CSE (Proteintech, San Diego, CA, USA), 1:2000 anti-Ferritin (Novous Biologicals, Etobicoke, ON, Canada), 1:2000 anti-GAPDH (Novous Biologicals, Etobicoke, ON, Canada), 1:2000 anti-NCO4A (Novous Biologicals, Etobicoke, ON, Canada), 1:2000 anti-NRF2 (Novous Biologicals, Etobicoke, ON, Canada), 1:2000 anti-Lamin A/C, 1:2000 p-eIF2α (Cell Signaling Technology), 1:2000 anti-eIF2α (Cell Signaling Technology, Danver, MA, USA), 1:2000 anti-αtubulin (Cell Signalling Technology, Danver, MA, USA), 1:1000 anti-cleaved caspase 3 (Cell Signalling Technology, Danver, MA, USA), 1:2000 anti-ATF4 (Thermofisher, Mississauga, ON, Canada), 1:2000 anti-p-AMPK (Cell Signaling Technology, Danver, MA, USA), 1:2000 anti-AMPK (Cell Signaling Technology, Danver, MA, USA), and 1:2000 LC3B (Novous Biologicals, Etobicoke, ON, Canada), were incubated in separate experiments for one and a half hours at room temperature. Wash steps were performed with PBS with 0.1% tween or TBS with 0.1% tween. HRP-conjugated goat anti-rabbit (1:2000) or rabbit anti-mouse (1:2000) antibodies were used as secondary antibodies and were incubated for one and a half hours at room temperature. Detection was performed using ECL (Cytiva, Montreal, QC, Canada) or Super Signal West Pico Plus (Thermofisher, Mississauga, ON, Canada) and imaged using Microchemi imaging systems (Froggabio, Concord, ON, Canada).

### 2.17. Quantitative PCR

Total RNA was isolated using the Trizol (Sigma, Oakville, ON, Canada) method. Reverse transcription with 2 µg RNA to obtain cDNA was performed using the Superscript First Strand system (Invitrogen, Burlington, ON, Canada). *CSE* primers included the forward primer AGCGATTACACCACAAACCAAG and the reverse primer ATCAGCACCCAGAGCCAAAGG. Primers for *18S* rRNA included the forward primer AGTCCCTGCCCTTTGTACACA and the reverse primer CGATCCGAGGGCCTCACTA. Real-time PCR was performed using SYBR Green PCR master mix in an iCycler iQ5 (BioRad, Mississauga, ON, Canada) with iCycler optical system software (version 3.1). Cycle conditions included 95 °C for 1 min, followed by 38 cycles of 95 °C for 15 s, 61 °C for 20 s, and 72 °C for 30 s, during which time an image was taken. Standard melt curve analysis was performed between temperatures of 58 °C and 95 °C with 1 °C increments. Relative mRNA quantification was calculated using the arithmetic formula 2^−ΔΔCT^, where ΔCT is the difference between the threshold cycle of a given target cDNA and an endogenous reference 18S cDNA.

### 2.18. Statistical Analysis and Reproducibility

All data were expressed as means ± standard errors (SEMs). A *p*-value less than 0.05 was considered statistically significant. Experiments utilizing 96-well plates had 6 wells designated per group, and all data were taken from 3 plates recorded in 3 individual experiments. Protein from 3 plates (35 mm each) was pooled in 1 tube, representing 1 sample, and 3 samples per group were run on gels for Western blotting. Data from all Western blots were taken from 4 gels recorded in individual experiments. For imaging experiments, 3 random fields were chosen from 6 plates (35 mm each) per group. Unpaired two-tailed Student’s *t*-tests were employed for comparisons between two groups, as appropriate. For analyses involving three or more groups, either one-way or two-way analysis of variance (ANOVA) was conducted, followed by Tukey’s multiple-comparison post hoc test.

## 3. Results

### 3.1. Iron Loading Decreased Viability and Caused Iron Overload in CSE-KO Cells

**Figure 1:**
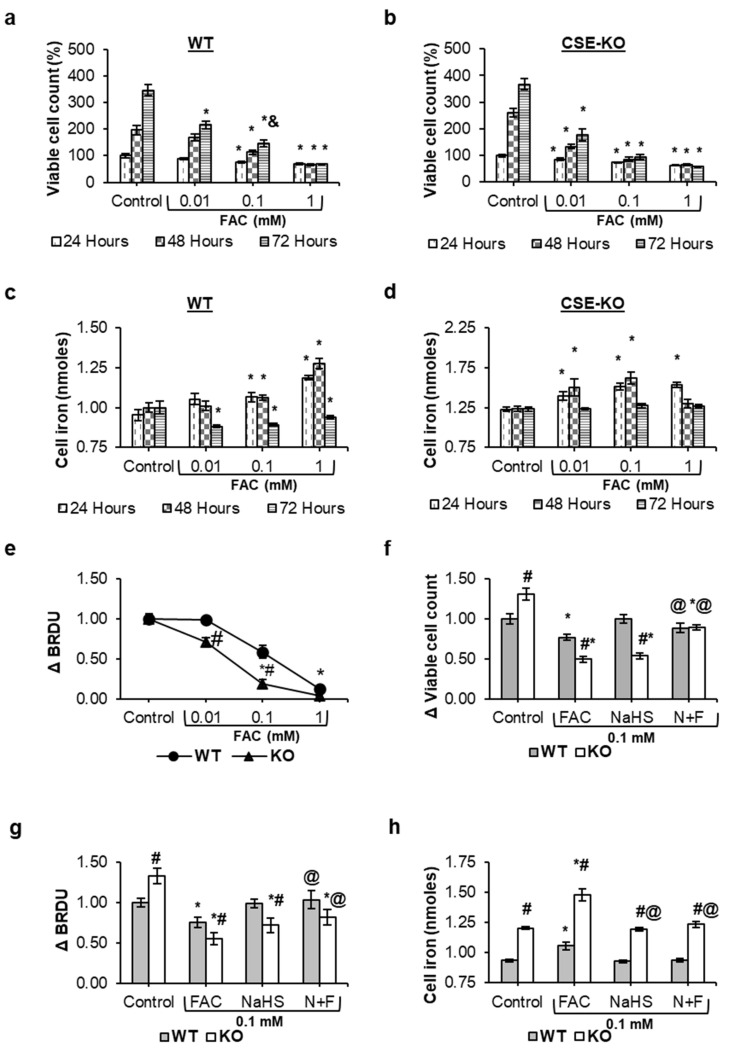
Effects of FAC on cell viability, iron concentration, and proliferation in cultured VSMCs. (**a**,**b**) Cell viability measured via MTT assay relative to controls of the same genotype (WT or CSE-KO). (**c**,**d**) Cellular iron concentrations. (**e**) Changes in VSMC proliferation relative to controls of the same genotype measured via BRDU assay after being exposed to FAC for 48 h. (**f**) Interaction effect of FAC and NaHS on cell viability measured with MTT assay. (**g**) Interaction effect of FAC and NaHS on cell proliferation measured with BRDU assay. (**h**) Interaction effect of FAC and NaHS on cellular iron concentrations measured with ferrozine assay. In (**f**–**h**), WT and CSE-KO cells were exposed to FAC for 48 h with or without NaHS pretreatment (N+F), and data were normalized to WT controls. * *p* < 0.05 vs. control group with the same genotype, & *p* < 0.05 vs. 24 h group with the same treatment, # *p* < 0.05 vs. WT group with the same treatment, @ *p* < 0.05 vs. FAC treatment with the same genotype group; *n* = 6, with 3 experimental repeats.

Iron loading with FAC significantly decreased the viability of both WT and CSE-KO cells (Figure 1). WT cells demonstrated greater iron tolerance than KO cells, as the viability of WT cells remained stable when exposed to 0.01 mM FAC for up to 48 h, whereas KO cells exhibited a significant viability decline within 24 h (Figure 1a,b). At 0.1 mM FAC, WT cells showed a time-dependent recovery, with viability increasing significantly after 72 h compared to the 24 h incubation. In contrast, KO cells displayed no such adaptive response, maintaining significantly reduced viability at all incubation periods relative to untreated controls.

The ferrozine assay demonstrated a linear increase in cellular iron concentration in both WT and KO cells, with the maximal iron concentration reached within 24 h of exposure to ferric ammonium citrate (FAC) (Figure 1c,d). In WT cells, treatment with 0.1 mM and 1 mM FAC significantly elevated the cellular iron concentration compared to untreated controls at 24 and 48 h. A sharp decline in intracellular iron was observed in WT cells by 72 h across all FAC concentrations. KO cells exhibited a similar initial trend, with iron concentrations peaking at 24 h. However, KO cells displayed a significant reduction in iron concentration by 72 h compared to earlier timepoints (Figure 1d), mirroring the decline seen in WT cells but with no recovery phase.

Distinct iron-handling dynamics between WT and KO cells emerged at the 0.01 mM FAC concentration. While WT cells exhibited no measurable change in iron concentration over 24–48 h, KO cells displayed a significant increase in iron accumulation within 24 h, sustaining elevated levels through 48 h (Figure 1c,d). The viability of WT cells remained unchanged with 0.01 mM FAC exposure for 24–48 h. In contrast, KO cells suffered a significant viability drop under the same conditions, correlating with their dysregulated iron accumulation. With 0.1 mM FAC, KO cells exhibited exacerbated iron loading and a steeper viability decline compared to WT cells.

With the highest concentration of FAC (1 mM), WT cells maintained an increased cellular iron concentration for 48 h, while KO cells failed to sustain iron levels beyond 24 h. These findings collectively underscore WT cells’ iron tolerance, mitigating iron toxicity and sustaining cell viability. The inability of KO cells to survive beyond 48 h in 0.1 mM FAC, coupled with the increase in intracellular iron accumulation within this timeframe, suggests that cell damage or death occurred. It is also interesting to note that KO cells have a higher viability and proliferation rate, as reported in our previous studies [15,24]. KO cells after 48 h of incubation without FAC treatment almost tripled their viable cell numbers in comparison to those at 24 h. In contrast, the viable cell numbers of the WT only doubled after 48 h of incubation as compared to 24 h of incubation. At 72 h, both WT and KO cells were overconfluent due to the restricted growth space, which is why a significant difference between the viability of WT and KO cells within the control group at this timepoint was not apparent. In all the following studies, 48 h was selected as the incubation time for the comparison of WT and KO cells under different conditions.

BrdU incorporation assays showed that FAC treatment at all concentrations significantly suppressed the proliferation of KO cells. Incubation with 0.01 mM FAC caused no significant change in WT proliferation, while 0.1 mM FAC induced a less severe decline compared to KO cells (Figure 1e). Next, exogenous H_2_S was introduced to KO cells using NaHS, an H_2_S salt, to determine whether H_2_S mitigates the excessive iron accumulation and restores cell viability and proliferation in FAC-treated KO cells. Pretreating KO cells with 0.1 mM NaHS, followed by adding 0.1 mM FAC, improved cell viability and proliferation as compared to the FAC-alone group (Figure 1f,g). NaHS treatment effectively reduced FAC-induced increase in cellular iron levels in both WT and KO cells (Figure 1h).

### 3.2. H_2_S Promotes Ferritin Upregulation

**Figure 2:**
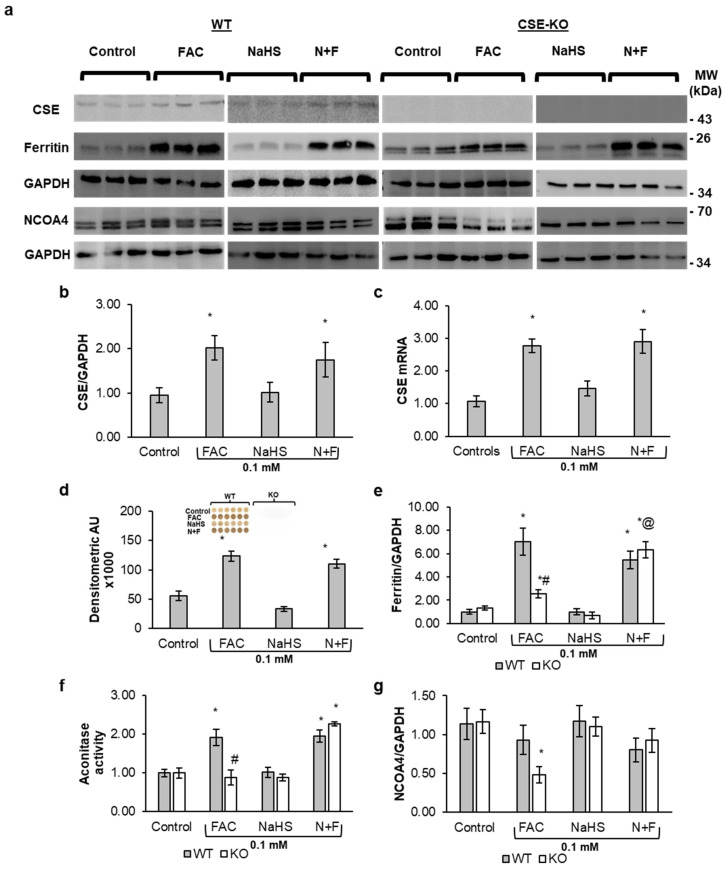
The effects of FAC on the CSE-H_2_S pathway and Ferritin expression. (**a**) Representative Western blots of CSE, Ferritin, and NCOA4 in WT and CSE-KO VSMCs. (**b**) Relative expression levels of CSE proteins in WT cells with different treatments. (**c**) Relative *CSE* mRNA levels in WT VSMCs with different treatments. (**d**) H_2_S production in WT and CSE-KO VSMCs with different treatments. (**e**) Relative expression levels of Ferritin in WT and CSE-KO cells. (**f**) Aconitase activities in different groups of VSMCs. (**g**) Relative expression levels of NCOA4 proteins in different groups of VSMCs. The treatment time for all groups was 48 h. * *p* < 0.05 vs. untreated control group with the same genotype, # *p* < 0.05 vs. WT group with the same treatment, @ *p* < 0.05 vs. 0.1 mM FAC with the same genotype; *n* = 3, with 4 experimental repeats. N+F, 0.1 mM NaHS pretreatment + 0.1 mM FAC.

CSE expression at both the protein and mRNA levels was increased in WT cells treated with FAC alone and the N+F group as compared to the control and NaHS-alone groups (Figure 2a–d). KO cells had no band for the CSE proteins with Western blotting, confirming their KO status (Figure 2a). Ferritin protein levels were increased in both WT and KO cells for the FAC-alone and N+F groups as compared to the control and NaHS groups (Figure 2a,e). Western blot results show that FAC-treated WT cells exhibited 2.8-fold higher Ferritin protein contents than FAC-treated KO cells (Figure 2e). Similarly, the presence of H_2_S affects Ferritin levels, as can be observed in Figure 2e. Adding 0.1 mM FAC with or without NaHS significantly increased H_2_S production in WT cells. Furthermore, no H_2_S production was detected in KO cells with all treatments aforementioned using the lead acetate paper method. This confirms that H_2_S production under the control condition and with FAC treatment was catalyzed by CSE.

Since Ferritin has antioxidant properties and sequesters free iron [25], it is possible that the increase in CSE expression and H_2_S level may increase iron sequestration via directly or indirectly increasing Ferritin expression. There was a significant, greater than six-fold, increase in Ferritin expression in the FAC-alone and N+F groups of WT cells compared to the control (Figure 2e). In contrast, KO cells showed a meager 2-fold increase in Ferritin expression with FAC treatment alone but a 6-fold increase in the N+F group, where the lack of endogenous H_2_S was compensated by exogenous NaHS.

Intracellular iron is primarily regulated via iron regulatory proteins 1 and 2 (IRP1 and IRP2) [26]. C-aconitase is another form of IRP1. The difference in aconitase activity between WT and KO VSMCs is summarized in Figure 2f. FAC treatment alone increased aconitase activity in WT cells but failed to do so in KO cells. However, both KO and WT cells show increased aconitase activity in the N+F groups.

Nuclear Receptor Coactivator 4 (NCOA4) is a selective cargo receptor that mediates ferritinophagy, which is the breakdown of Ferritin via autophagy to release iron in the cell, often leading to iron overload and ferroptosis [27,28]. Figure 2g shows a sharp decrease in NCOA4 in FAC-treated KO cells. This suggests that the lower Ferritin level in KO cells resulted from impaired Ferritin expression rather than breakdown of already expressed Ferritin via ferritinophagy.

### 3.3. H_2_S Inhibits Iron-Mediated Oxidative Stress

**Figure 3:**
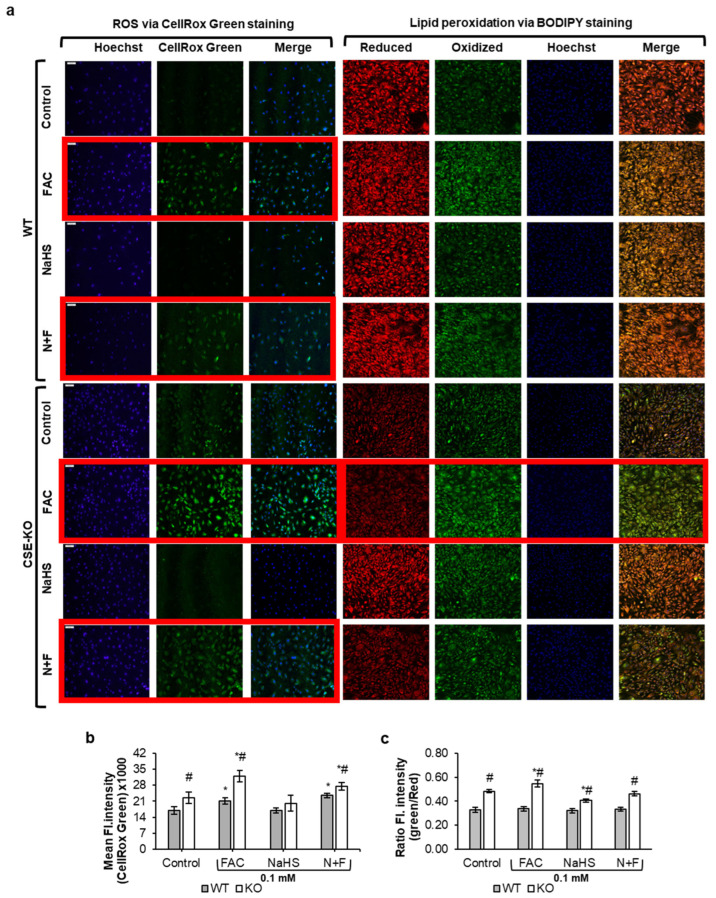
The effects of H_2_S on iron-mediated oxidative stress and lipid peroxidation. (**a**) Representative images of oxidative stress measured via CellRox green and lipid peroxidation via BODIPY 581/591 C11 staining in WT and CSE KO cells with different treatments for 48 h. (**b**) Mean fluorescence intensity of CellRox Green normalized to Hoechst. (**c**) Ratio of mean fluorescent intensity in the green (oxidized BODIPY) and red (reduced BODIPY) channels for BODIPY dye. Groups with significant differences are in a red border. * *p* < 0.05 vs. control with the same genotype, # *p* < 0.05 vs. WT group with the same treatment. From each 35 mm plate, 3 random fields were chosen, 18 images per group. Scale bar represents 100 µm. Control, PBS treatment; N+F, 0.1 mM NaHS pretreatment + 0.1 mM FAC.

Elevated ROS levels were detected in the FAC and N+F groups of both WT and KO cells (Figure 3a,b). However, only the FAC-treated KO cells, not the WT cells, exhibited significant lipid peroxidation as compared to the controls (Figure 3c). The absence of endogenous H_2_S in KO cells appears to lead to ROS accumulation to an extent that triggers lipid peroxidation. The addition of NaHS lowered lipid peroxidation in FAC-treated KO cells (Figure 3c, N+F group) as compared to the FAC-alone and control groups.

### 3.4. Iron-Treated CSE-KO Cells Undergo Apoptosis

**Figure 4:**
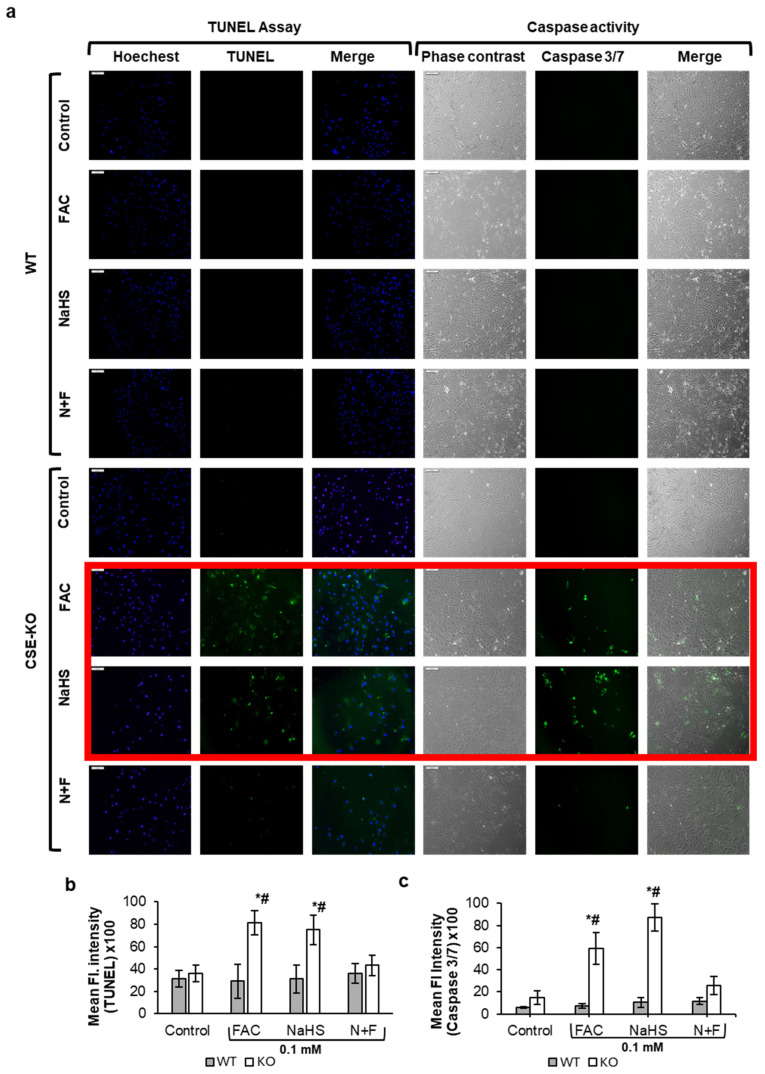
The effects of H_2_S on iron-mediated apoptosis. (**a**) Representative images of apoptosis measured via TUNEL and CellEvent caspase 3/7 green staining in WT and CSE KO cells. (**b**) Mean fluorescence intensity of TUNEL-positive cells normalized to Hoechst. (**c**). Mean fluorescent intensity in the green channels for CellEvent caspase 3/7 dye. Groups with significant differences are in a red border. * *p* < 0.05 vs. control with the same genotype, # *p* < 0.05 vs. WT group with the same treatment. From each 35 mm plate, 3 random fields were chosen, 18 images for each group. Scale bar represents 200 µm. Control, PBS treatment; N+F, 0.1 mM NaHS pretreatment + 0.1 mM FAC.

**Figure 5:**
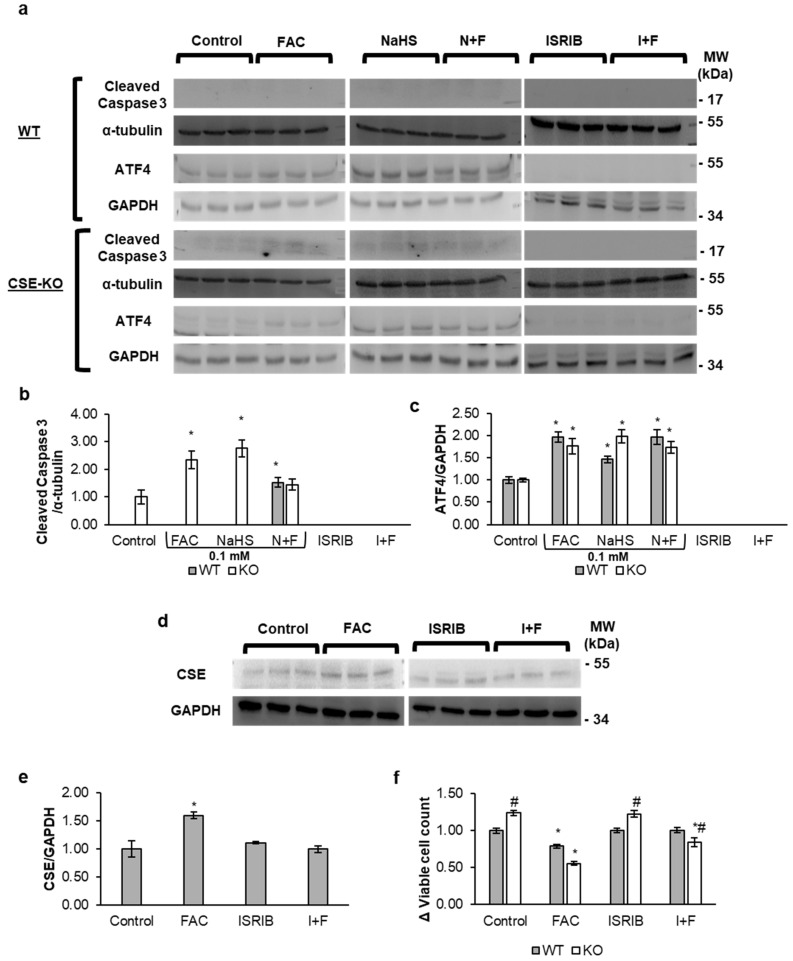
Iron upregulates CSE/H_2_S via ATF4, which in turn prevents apoptosis. (**a**) Representative Western blots of cleaved caspase 3, α-tubulin, and ATF4 in WT and CSE-KO VSMCs. (**b**) Relative expression of cleaved caspase 3 proteins. (**c**) Relative expression of ATF4 proteins. (**d**) Representative Western blots of CSE in WT cells. (**e**) Relative expression of CSE proteins in WT cells. (**f**) Viable cell count measured via MTT assay. All treatments were of 48 h duration. * *p* < 0.05 vs. untreated control group, # *p* < 0.05 vs. similar WT group. *n* = 3, with 4 experimental repeats. Control, PBS treatment; N+F, 0.1 mM NaHS pretreatment + 0.1 mM FAC; ISRIB at 0.1 µM was used; I+F, 0.1 µM of ISRIB pretreatment + 0.1 mM FAC.

The results from the TUNEL assay (Figure 4a,b) showed increased DNA fragmentation in CSE-KO cells treated with FAC or NaHS only. There was no observable fragmentation in WT cells with any referred treatment. Higher caspase activity was found in KO cells treated with FAC or NaHS alone as compared to controls (Figure 4c). However, CSE-KO cells with the N+F treatment had significantly lower caspase activity than those with the FAC-alone treatment, indicating that exogenous H_2_S can lower caspase activity via its interaction with FAC. Moreover, Western blot analysis showed a significant increase in cleaved caspase 3, the active form of caspase, in FAC- and NaHS-treated KO cells (Figure 5a,b). No significant caspase activity was observed in all groups of WT cells, as determined by caspase 3/7 staining. Interestingly, Western blot analysis did not detect cleaved caspase 3 bands in the control, FAC, or NaHS groups. Although faint bands appeared in the N+F group of WT cells, no apoptosis of these cells could be confirmed due to lack of DNA fragmentation and caspase activity. This confirms that CSE-KO cells undergo apoptosis after increased iron overload and oxidative stress as compared to WT cells.

### 3.5. Iron Upregulates CSE Expression via the ROS-eIF2α-ATF4 Pathway

**Figure 6:**
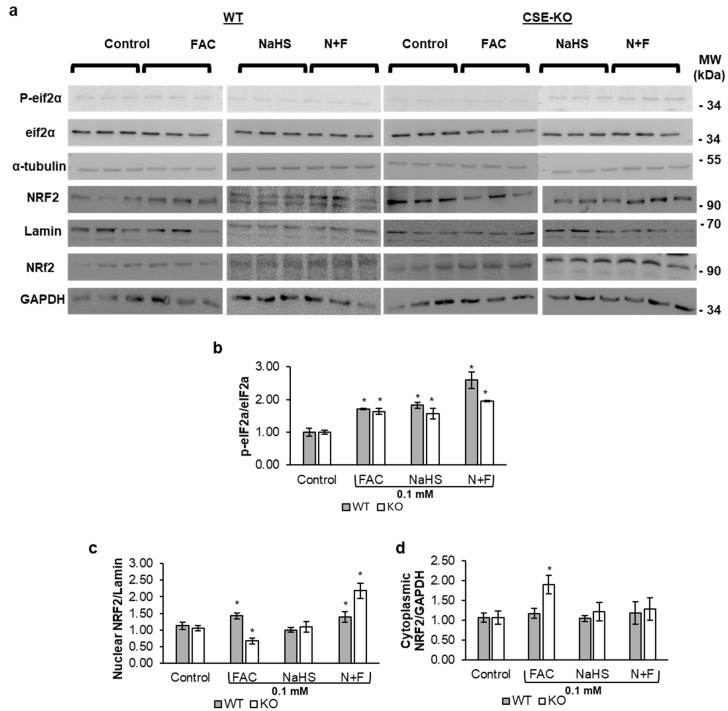
Iron activates the integrated stress response and increases nuclear translocation of NRF2. (**a**) Representative Western blots of NRF2 from nuclear fractions (Lamin) and cytoplasmic fractions (GAPDH) and p-eIF2α and eIF2α in WT and CSE-KO VSMCs. (**b**) Phosphorylation of eIF2α. (**c**) Relative expression of nuclear NRF2. (**d**) Relative expression of cytoplasmic NRF2. * *p* < 0.05 vs. control group with the same genotype. *n* = 3, with 4 experimental repeats. All cells received the treatments for 48 h. Control, PBS treatment; N+F, 0.1 mM NaHS pretreatment + 0.1 mM FAC.

We further investigated the mechanism whereby iron increases CSE expression. The Western blot data in Figure 5c show that FAC increased ATF4 expression as compared to the controls in both WT and KO cells. ISRIB is a blocker of ATF4 and the integrated stress (ISR) pathway. Figure 5c–e show that the addition of ISRIB inhibited ATF4 expression in the ISRIB-only and I+F groups. ISRIB also blunted the iron-mediated increase in CSE expression in FAC-treated WT cells (I+F). This confirmed that the iron-mediated increase in CSE occurred via ATF4. Interestingly, ISRIB-pretreated KO cells in FAC had no cleaved caspase 3 activation as compared to FAC-treated KO cells (Figure 5b). ISRIB and FAC co-treatment of KO cells showed an increase in cell viability compared to KO cells treated with FAC alone (Figure 5f). Figure 6a,b show an increase in the p-eIF2α-to-eIF2α ratio in FAC-treated WT and KO cells. The increase in ROS, expression of ATF4, and phosphorylation of eIF2α confirm that iron triggers ISR in both WT and KO cells.

### 3.6. H_2_S Increases Nuclear Translocation of NRF2 in Response to ISR

NRF2 expression was elevated in FAC-treated WT and KO cells (Figure 6a–d), indicating that NRF2 is upregulated in iron-treated VSMCs irrelative to the presence of CSE. In WT cells, this increase was observed in nuclear fractions for both the FAC and N+F groups. In KO cells, however, NRF2 upregulation in the FAC group was confined to the cytoplasmic fractions, whereas in the N+F group, NRF2 upregulation was evident in the nuclear fractions. These results suggest that exogenous H_2_S facilitates NRF2 nuclear translocation in FAC-treated KO cells.

### 3.7. H_2_S Protects Against Iron-Mediated Oxidative Stress via Upregulating AMPK-Mediated Autophagy

**Figure 7:**
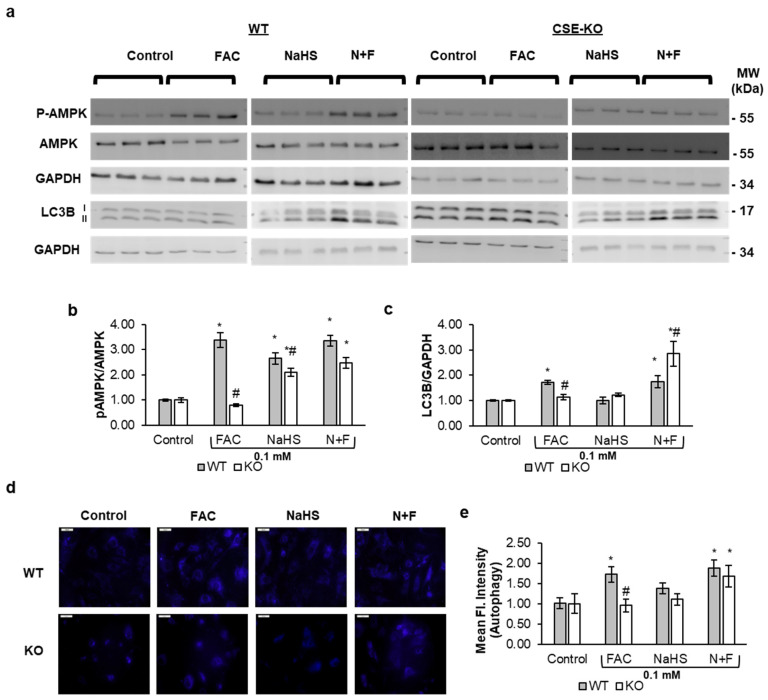
The interaction effect of iron and CSE/H_2_S on VSMC autophagy. (**a**) Representative Western blots of p-AMPK and LC3B in WT and CSE-KO VSMCs. (**b**) Relative levels of p-AMPK proteins. (**c**) Relative expression levels of LC3B proteins. (**d**) Representative images of autophagosome staining in treatment groups. (**e**) Mean fluorescent intensity representing autophagosome activity. The treatment duration for all groups of cells was 48 h. Scale bar represents 50 µm. Control, PBS treatment; N+F, 0.1 mM NaHS pretreatment + 0.1 mM FAC. * *p* < 0.05 vs. control. # *p* < 0.05 vs. WT group with the same treatment. n = 3, with 4 experimental repeats.

Western blot results showed increased AMPK phosphorylation in FAC-treated WT cells but not in FAC-treated KO cells (Figure 7a,b). However, both WT and KO cells with N+F treatments exhibited increased phosphorylation of AMPK. This shows that endogenous and exogenous H_2_S can lead to an increase in p-AMPK in iron-treated VSMCs. Further, the increased LC3B expression (Figure 7c) and increased autophagosome activity (Figure 7d,e) also indicated that autophagy is significantly higher in FAC-treated WT cells and NaHS-pretreated WT and KO cells. FAC-treated KO cells did not show significant LC3B expression (Figure 7c), nor any measurable autophagosome activity (Figure 7d,e). This shows that endogenous or exogenous H_2_S also upregulates autophagy as a response to iron-catalyzed ISR via ROS.

## 4. Discussion

The interplay between iron accumulation, oxidative stress, and H_2_S metabolism constitutes a complex regulatory network critical for iron homeostasis and cell survival. Some previous studies examined the relationship between iron and H_2_S by using toxic levels of extracellular iron to induce iron overload [10,29]. Rather than using inorganic sources of iron like FeCl_2_, FeCl_3_, or other inorganic iron salts [10,30,31], the current study used FAC because 0.1 mM FAC contains 17 µM of iron and 100 µM of citrate, which quantities are in the physiological ranges of iron (17–23 µM) and citrate (100–130 µM) in mouse plasma [2,20]. FAC closely mimics the physiological entry of iron into cells, where iron is weakly complexed with citrate [2,32,33]. In biological systems, non-transferrin-bound iron (NTBI), often termed “labile iron,” exists in loose complexes with molecules like citrate or ascorbate [2,34,35]. Under normal conditions, blood iron is either tightly bound to transferrin or loosely associated with citrate, with no detectable free ferric (Fe^3+^) or ferrous (Fe^2+^) ions [2]. During systemic iron overload, citrate-bound iron gains significance as the primary form facilitating cellular uptake, while inorganic iron forms remain physiologically irrelevant due to their absence in biological environments. Our findings revealed that FAC at physiologically or pathophysiologically relevant concentrations significantly reduced the viability and proliferation of both WT and CSE-KO cells, with a more pronounced effect observed in KO cells compared to WT counterparts. Pretreatment with 0.1 mM NaHS lowered cellular iron levels and rescued the FAC-induced decline in viability and proliferation in KO cells.

FAC-treated WT cells exhibited significantly lower intracellular iron concentrations compared to CSE-KO cells, a difference driven by H_2_S-mediated upregulation of ferroportin and Ferritin. While divalent metal transporter 1 (DMT1) facilitates iron import, ferroportin exports excess iron [2]. Building on prior findings by Zhu et al., H_2_S specifically upregulates ferroportin in iron-treated WT cells without altering DMT1 expression in either WT or KO cells [10]. This ferroportin induction enhances iron export, thereby reducing intracellular iron levels in WT cells [10]. Concurrently, endogenous H_2_S elevates Ferritin expression in WT cells, effectively sequestering labile iron and further diminishing intracellular iron concentrations, as demonstrated in this study. Together, these mechanisms underscore H_2_S’s dual role in mitigating iron overload through coordinated export and sequestration.

Our study further shows that FAC upregulated CSE expression and activity. Some previous studies did not observe increased CSE expression in iron-overload VSMCs or cardiac cells when they used 0.1 mM of FeCl_2_ for 24 h to induce iron overload [10,11]. FeCl_2_ at this concentration yields 0.1 mM ferrous iron, which is 5.88 times higher than the 17 µM concentration of ferric iron in 0.1 mM FAC. Iron concentrations and bioavailability significantly differ between the inorganic iron chlorides and FAC, with the latter showing higher bioavailability [32,33]. Additionally, the 24 h incubation period used in previous studies may not have been long enough to induce a detectable increase in CSE proteins, whereas in our study, CSE upregulation was observed after 48 h of FAC treatment.

The mechanism by which iron triggers the upregulation of CSE was investigated in the current study. It has been reported that four transcription factors can increase CSE expression: SP1, NRF2, ELK1, and ATF4 [36]. Iron overload has been shown to downregulate SP1, making SP1 an unlikely candidate for increasing CSE [37,38]. Our previous study showed that nuclear translocation of NRF2 occurs in WT cells but not in KO cells under oxidative stress [39], indicating the stimulatory role of endogenous H_2_S for NRF2 nuclear translocation. ELK1 activity in oxidative stress has been shown to occur downstream of NRF2 [40]. Thus, an increase in endogenous H_2_S could be a pre-requirement for ELK1 upregulation and NRF2 nuclear translocation. We have previously shown ATF4-increased CSE expression in response to Golgi stress in murine myoblast (C2C12) cells [41]. Moreover, ATF4 has been shown in different studies to be directly involved in ROS-mediated oxidative stress [42,43,44,45,46]. Sarcinelli et al. showed that ER stress leads to ROS-mediated oxidative stress, which upregulates NRF2 as a protective mechanism via upregulating PKR-like endoplasmic reticulum kinase, which phosphorylates eIF2α, leading to upregulation of ATF4 [46]. Zhang et al. showed that ER stress-related oxidative stress increased autophagy in mouse spermatocyte-derived cells via phosphorylation of eIF2α and upregulation of ATF4 [44]. The ER stress response has three arms, ATF4, inositol-requiring enzyme 1, and ATF6. Given that ATF4 is the primary regulator of ROS-related ER stress [47] and a transcription factor for CSE [36], our focus was directed toward ATF4 rather than the other pathways. The interaction of the CSE/H_2_S system with inositol-requiring enzyme 1 and ATF6 has been unknown. Our current study shows that ATF4 was upregulated in FAC-treated WT and KO cells via the ROS-mediated ISR. Pretreatment of WT VSMCs with the ATF4 inhibitor, ISRIB, abolished FAC-induced upregulation of CSE, pinpointing ATF4 as the pivotal transcription factor responsible for iron-induced CSE upregulation.

Ferritin stores iron in its redox-inactive form [48]. FAC increased Ferritin levels in both WT and KO cells. However, the increased Ferritin level in FAC-treated KO cells (2 folds) was significantly lower than in FAC-treated WT cells (6 folds), indicating that the stimulatory effect of iron on Ferritin expression is facilitated by endogenous H_2_S. Pretreating KO cells with NaHS followed with FAC increased Ferritin levels to a degree comparable to that in FAC-treated WT cells. Zhu et al. also found that iron (Fe^2+^)-treated CSE-KO cells had significantly lower Ferritin upregulation compared to iron-treated WT cells, but adding NaHS significantly improved Ferritin levels in iron-treated KO cells [10]. They further reported that in the presence of H_2_S, IRP1, which is bound to *ferritin* mRNA, is readily converted to cytosolic aconitase. This releases the *ferritin* mRNA, ready to be translated. Our results also showed the same phenomenon. Significant aconitase activity and Ferritin expression were observed in the FAC-treated WT and N+F groups of WT and KO cells, but not in FAC-treated KO cells.

The weaker Ferritin upregulation in KO cells compared to WT cells may also reflect impaired iron buffering via NCOA4-mediated ferritinophagy. Contrary to expectations, NCOA4 expression decreased in FAC-treated KO cells, suppressing ferritinophagy. As shown by Zhao et al., mitochondrial aconitase generates 3Fe-4S clusters under iron-replete conditions, which bind NCOA4 to trigger its degradation—a process distinct from cytosolic aconitase/IRP1 signaling [48,49,50]. In KO cells, elevated ROS likely activated mitochondrial aconitase to produce 3Fe-4S clusters, promoting NCOA4 degradation despite low Ferritin levels and iron overload. This suggests that NCOA4 downregulation serves as an adaptive response to excessive labile iron, further exacerbating dysregulation.

In parallel, ferroptosis—an iron-dependent, caspase-independent cell death pathway marked by lipid peroxidation [9,51,52,53,54]—emerged as a secondary mechanism in KO cells. At physiological iron levels (0.1 mM FAC), KO cells exhibited caspase-3 activation and TUNEL-positive apoptosis, aligning with studies linking iron dysregulation to mitochondrial lipid peroxidation [55,56]. The absence of CSE/H_2_S disrupted Ferritin-mediated iron sequestration, enabling gradual oxidative damage and apoptosis (Figure 1). While ferroptosis typically requires acute iron overload, partial viability rescue in KO cells despite caspase inhibition (ISRIB; Figure 5f) implied residual ferroptosis. At 1 mM FAC, both WT and KO cells showed rapid, caspase-independent death consistent with ferroptosis, mirroring Wang et al.’s findings in myoblasts [9]. Thus, H_2_S deficiency shifts iron-induced death from apoptosis (low iron) to ferroptosis (high iron), underscoring H_2_S’s role in maintaining iron homeostasis and suppressing oxidative cell death.

Oxidative stress is a common result of iron overload [2]. Hydrogen peroxide generated in mitochondria or cytoplasm can react with the excess free iron in labile iron pools of cells to produce ROS in toxic amounts via the Fenton reaction [2]. The excess ROS lead to oxidative stress and widespread cell damage. Our previous studies have shown that KO cells have higher basal ROS levels than WT cells due to the absence of CSE and high homocysteine levels in KO cells [8]. Our current study reaffirmed the higher ROS basal level in KO cells compared to WT cells (Figure 3a,b). Moreover, higher ROS levels were observed in the FAC and N+F groups as compared to controls, in both WT and KO cells. Interestingly, NaHS lowered basal ROS in KO cells, which confirmed our previous finding [57]. When the accumulation of ROS overwhelms the natural antioxidant capacity of the cell, oxidative stress occurs [58], leading to detrimental chain reactions, such as lipid peroxidation. While ROS production increased in both FAC-treated WT and KO cells, only the FAC-treated KO cells produced sufficient ROS to induce lipid peroxidation. It was also noticed that pretreating FAC-treated KO cells with NaHS significantly reduced lipid peroxidation compared to untreated FAC-treated KO cells. This suggests that, in the absence of endogenous H_2_S, iron-treated VSMCs experience a pronounced increase in oxidative stress, which can be effectively mitigated by the addition of exogenous H_2_S. Further, only FAC-treated KO cells exhibited DNA fragmentation detected with the TUNEL assay and caspase3/7 activity, which could be reversed by pretreating the KO cells with exogenous H_2_S. The effect of 0.1 mM NaHS on apoptosis has been reported [14]. Our previous studies showed that 0.1 mM NaHS decreases the viability and proliferation of KO cells but not WT cells [19]. KO cells are devoid of endogenous H_2_S and are more sensitive to apoptotic triggers as compared to WT cells, which require a much higher H_2_S concentration (0.5 mM) to induce significant apoptosis [14]. It is interesting to note that KO cells with N+F treatment exhibited a significant decrease in DNA fragmentation as compared to FAC-treated KO cells. This shows that exogenous H_2_S offers protection against iron-overload-mediated DNA fragmentation.

H_2_S mitigates iron-overload damage in FAC-treated WT cells by promoting the nuclear translocation of NRF2, a master regulator of antioxidant gene expression [39,59]. Mechanistically, H_2_S directly modifies Kelch-like ECH-associated protein 1 (KEAP1)—an NRF2 inhibitor—via S-sulfhydration, destabilizing the KEAP1-NRF2 complex and enabling NRF2 to translocate into the nucleus, as demonstrated in our prior work [39]. This activation of NRF2 drives the transcription of antioxidant genes, counteracting oxidative stress. In the current study, FAC-treated WT cells exhibited robust NRF2 nuclear accumulation, whereas KO cells showed cytoplasmic retention of NRF2, correlating with their heightened oxidative damage. Exogenous H_2_S supplementation rescued this deficit in KO cells, restoring NRF2 nuclear localization. This aligns with findings by Kasai et al. [60], who linked NRF2 activation to the ROS-p-eIF2α-ATF4 pathway during integrated stress responses. Thus, H_2_S orchestrates a dual protective mechanism: direct KEAP1 S-sulfhydration to liberate NRF2 and indirect amplification of antioxidant defenses via NRF2-driven gene expression. This pathway not only buffers iron-induced oxidative stress but also highlights H_2_S’s central role in maintaining redox homeostasis.

H_2_S critically modulates the balance between autophagy and apoptosis in vascular smooth muscle cells. In FAC-treated WT cells, H_2_S enhances AMPK phosphorylation, driving autophagy via mTORC1/ULK1/PIK3C3 pathways [61,62,63,64], while suppressing apoptosis through reduced cleaved caspase-3. Conversely, CSE-KO cells under FAC exhibit AMPK inactivation, failed autophagy, and elevated apoptosis—phenotypes rescued by exogenous H_2_S. This aligns with studies linking AMPK suppression to apoptosis in stressed VSMCs [13,65,66]. The interplay extends to iron–H_2_S dynamics: iron overload upregulates CSE, boosting H_2_S to sequester labile iron into Ferritin, thereby mitigating oxidative damage. In CSE-KO cells, excess iron triggers ATF4-mediated ISR, activating caspases and apoptosis [67,68]. WT cells, however, leverage H_2_S to activate AMPK, counterbalancing ATF4’s pro-apoptotic effects. ISRIB inhibition of ATF4 in KO cells prevents caspase activation, underscoring ATF4’s dual role as both an apoptotic driver and CSE upregulator. Thus, H_2_S orchestrates survival by coupling iron detoxification (via Ferritin) to AMPK-driven autophagy, while its absence shifts the balance toward ATF4-caspase apoptosis. These findings position H_2_S as a molecular fulcrum, harmonizing iron metabolism and stress responses to prioritize cell survival. A detailed overview of the proposed mechanism is presented in the graphical abstract, summarizing the key interactions explored in this study.

H_2_S-mediated autophagy may play an important role in cellular homeostasis beyond apoptosis. For instance, in senescence, H_2_S can mitigate oxidative stress and promote cellular longevity, potentially delaying aging-related dysfunction. Additionally, its influence on autophagy may affect cell proliferation by modulating metabolic pathways and nutrient availability, impacting tissue regeneration and repair. These broader effects highlight the need for further investigation into H_2_S’s role in complex cellular processes.

We acknowledge that our study utilized isolated VSMCs to examine the direct interaction between free iron and H_2_S within the cell. This interaction is likely more complex in vivo, where hormones, signaling molecules, transferrin-bound iron, and hepcidin may influence the process. However, our findings provide a foundational basis for future research exploring the interaction between iron and H_2_S in more intricate in vivo environments.

Our findings hold key implications for diseases like type 2 diabetes, where low blood H_2_S levels [69] and iron overload [70] coexist. H_2_S-based interventions, such as H_2_S donors; dietary restriction [71] to boost endogenous H_2_S production; and the application of metformin, which elevates tissue H_2_S [72,73] and lowers iron via AMPK [72], may mitigate iron-caused pathological damage. Metformin’s iron-lowering effects may hinge on H_2_S, as our study identifies CSE as being central to AMPK activation under iron stress. Low H_2_S levels also correlate with cerebrovascular diseases (e.g., Alzheimer’s), where iron exacerbates progression [74]; restoring H_2_S could slow such decline. In hypertension, iron overload’s vascular risks may amplify without CSE/H_2_S [2]. This synergy suggests that patients with low levels of endogenous H_2_S face heightened iron-related damage, warranting strategies to elevate H_2_S (e.g., dietary/metformin therapies). Our work underscores the need to clinically explore H_2_S–iron interplay for therapeutic breakthroughs.

## 5. Conclusions and Perspectives

Reduced endogenous H_2_S production in VSMCs disrupts iron homeostasis, causing iron overload at physiological or pathological extracellular iron levels via impaired ferroportin-mediated export and diminished Ferritin sequestration. This disruption tilts the apoptosis–autophagy balance toward apoptosis. In contrast, H_2_S safeguards VSMCs by enhancing iron export, upregulating Ferritin to buffer excess iron, activating NRF2 antioxidant pathways, and promoting AMPK-dependent autophagy. Collectively, these actions inhibit apoptosis and reduce vascular injury, underscoring H_2_S’s pivotal role in iron regulation. These insights highlight H_2_S-boosting therapies as promising strategies for iron-related vascular pathologies.

## Data Availability

The original contributions presented in this study are included in the article. Further inquiries can be directed to the corresponding author.

## Abbreviations

AMPK	Adenosine monophosphate-activated protein kinase
ANOVA	Analysis of variance
ATF4	Activating transcription factor 4
BrdU	Bromodeoxyuridine
CSE	Cystathionine gamma-lyase
DMEM	Dulbecco’s Modified Eagle Medium
DMSO	Dimethyl sulfoxide
eIF2α	Eukaryotic translation initiation factor 2
FAC	Ferric ammonium citrate
IRP1	Iron regulatory proteins 1
ISR	Integrated stress response
ISRIB	Integrated stress response inhibitor
KEAP1	Kelch-like ECH-associated protein 1
NCOA4	Nuclear Receptor Coactivator 4
NRF2	Nuclear factor erythroid 2-related factor 2
PBS	Phosphate-buffered saline
ROS	Reactive oxygen species
TBS	Tris-buffered saline
VSMCs	Vascular smooth muscle cells
